# Cardiac myosin-binding protein C in ST-elevation myocardial infarction

**DOI:** 10.1093/ehjacc/zuag032

**Published:** 2026-03-25

**Authors:** Ramyah Rajakulasingam, Bashir Alaour, Sam McGrath, Liam S Couch, Yamam Sbeih, Zohya Khalique, Emanuela Falaschetti, Alexander Tindale, Emily-Jane Cantor, Catherine J Beattie, Miles Dalby, Dudley J Pennell, Michael Marber, Ranil de Silva

**Affiliations:** National Heart and Lung Institute, Imperial College London, Sydney Street, London SW3 6NP,UK; Royal Brompton and Harefield Hospitals, Guy’s and St Thomas’ NHS Foundation Trust, London, UK; King’s BHF Centre of Research Excellence, King’s College London, London, UK; King’s BHF Centre of Research Excellence, King’s College London, London, UK; King’s BHF Centre of Research Excellence, King’s College London, London, UK; King’s BHF Centre of Research Excellence, King’s College London, London, UK; National Heart and Lung Institute, Imperial College London, Sydney Street, London SW3 6NP,UK; Royal Brompton and Harefield Hospitals, Guy’s and St Thomas’ NHS Foundation Trust, London, UK; Imperial Clinical Trials Unit, Imperial College London, London, UK; National Heart and Lung Institute, Imperial College London, Sydney Street, London SW3 6NP,UK; Royal Brompton and Harefield Hospitals, Guy’s and St Thomas’ NHS Foundation Trust, London, UK; National Heart and Lung Institute, Imperial College London, Sydney Street, London SW3 6NP,UK; Royal Brompton and Harefield Hospitals, Guy’s and St Thomas’ NHS Foundation Trust, London, UK; Royal Brompton and Harefield Hospitals, Guy’s and St Thomas’ NHS Foundation Trust, London, UK; Royal Brompton and Harefield Hospitals, Guy’s and St Thomas’ NHS Foundation Trust, London, UK; National Heart and Lung Institute, Imperial College London, Sydney Street, London SW3 6NP,UK; Royal Brompton and Harefield Hospitals, Guy’s and St Thomas’ NHS Foundation Trust, London, UK; King’s BHF Centre of Research Excellence, King’s College London, London, UK; National Heart and Lung Institute, Imperial College London, Sydney Street, London SW3 6NP,UK; Royal Brompton and Harefield Hospitals, Guy’s and St Thomas’ NHS Foundation Trust, London, UK

**Keywords:** Cardiac myosin-binding protein C, High-sensitivity cardiac troponin I, ST-elevation myocardial infarction, Myocardial infarction size, Microvascular obstruction, Myocardial injury

## Abstract

**Aims:**

Cardiac myosin-binding protein C (cMyC) is a novel biomarker of myocardial injury, rising and falling more rapidly than cardiac troponins in myocardial infarction (MI), potentially enabling earlier diagnosis. Its performance has not been assessed in reperfused acute ST-segment elevation myocardial infarction (STEMI), against gold-standard biochemical (high-sensitivity cardiac troponin I, hs-cTnI) or imaging (cardiovascular magnetic resonance, CMR) biomarkers. This study tested the hypotheses that: i) cMyC correlates with acute and final MI size by late gadolinium enhancement (LGE) CMR and ii) cMyC is related to the presence of acute microvascular obstruction (MVO) by CMR.

**Methods and results:**

Blood samples were obtained at 6 ± 2 hourly intervals for 24 hours (hrs) for measurement of hs-cTnI and cMyC concentrations in patients with reperfused acute STEMI. Patients underwent 3T LGE-CMR at ∼3–5 days (n = 69) and ∼4 months (n = 65) after reperfusion. Acute cMyC at all timepoints significantly correlated with acute and final MI size on LGE-CMR, most strongly at 6-hrs post reperfusion (r = 0.7, P < 0.001). cMyC at 6-, 12-, 18− and 24-hrs demonstrated significant discriminatory power in identifying patients with acute MVO, with the 6-hr level having the highest discriminative power. Hs-cTnI correlated more strongly with acute and final MI size compared with cMyC and had significantly higher discriminatory ability in identifying MVO at 12−, 18− and 24-hrs.

**Conclusion:**

cMyC is a quantitative biochemical biomarker of myocardial injury in reperfused STEMI. Further studies, using optimised high-sensitivity assays, are warranted to evaluate its potential as a novel biomarker after acute MI.

Key points

- Hs-cTnI remains the current reference standard for biochemical estimation of MI size and predictor of MVO.
- cMyC is additionally a quantitative biochemical biomarker of acute and final MI size determined by LGE-CMR and has significant discriminatory power in identifying high clinical risk patients with acute MVO.
- cMyC demonstrates the strongest correlations with MI size and ability to identify acute MVO at the 6-hr sampling timepoint post reperfusion.

## Introduction

Cardiac myosin-binding protein C (cMyC) is a promising clinically useful biomarker of myocardial injury, which rises and falls more rapidly than cardiac troponins in experimental myocardial infarction (MI),^1^ potentially enabling earlier diagnosis of acute MI. The performance of cMyC as a quantitative biomarker of myocardial injury has not been assessed in reperfused acute ST-segment elevation myocardial infarction (STEMI), against gold standard biochemical (high-sensitivity cardiac troponin I, hs-cTnI) or imaging (cardiovascular magnetic resonance, CMR) biomarkers. In a prospective single centre cohort study, we tested the hypotheses that: (i) cMyC correlates with acute and final MI size by late gadolinium enhancement (LGE)-CMR and (ii) cMyC is related to the presence of acute microvascular obstruction (MVO) by CMR.

## Methods

Patients with first presentation STEMI and single vessel disease on index angiography undergoing primary percutaneous coronary intervention (PPCI) were prospectively enrolled (September 2019–November 2021). The study population was described previously.^2^

Blood samples were obtained at arterial sheath insertion, and then at 6 ± 2 hourly intervals for 24 hours (hrs) for measurement of hs-cTnI and cMyC concentrations. Serum was stored at −80°C for batch cMyC analysis of duplicate aliquots using an in-house electrochemiluminescence sandwich enzyme-linked immunosorbent assay using high-affinity N-terminal capture and detection monoclonal antibodies. The 99th percentile upper reference limit (URL) for cMyC is 87 ng/L. Limit of detection (LoD) is 14 ng/L (CV 12%) with a detection range up to 80 000 ng/L. Hs-cTnI was measured using Beckman Coulter Access assay,^3^ for which the 99th percentile URL is 17.5 ng/L and 10% CV limit of quantification (LoQ) is 5.6 ng/L.

Patients underwent 3T CMR (Magnetom Vida, Siemens, Erlangen) at ∼3–5 days and ∼4 months after PPCI. LGE quantification of MI size was performed using the full-width-at-half-maximum method on the short axis stack (cvi42 software Version 5.13.3, Circle Cardiovascular Imaging, Canada)^4^ and expressed as infarct volume (mL). MVO was defined as a dark zone on early gadolinium enhancement imaging, which remained present within an area of LGE at 10–15 min post contrast injection.^5^ The study was approved by the London–Harrow Research Ethics Committee (19/LO/0434).

Data are reported as median [IQR]. Statistical analyses were performed using IBM SPSS Statistics (version 28.0) and Stata (version 16). Correlations between serum biomarkers and CMR parameters were performed using Spearman's rank test. Receiver operating characteristic (ROC) curve analysis was applied to determine the area under the curve (AUC) for identification of acute MVO for all serum biomarkers with comparison of AUCs by DeLong's test^6^; the optimal cut-off values were determined via the Youden index for variables with significant discriminatory power (P < 0.05).

## Results

**Figure 1:**
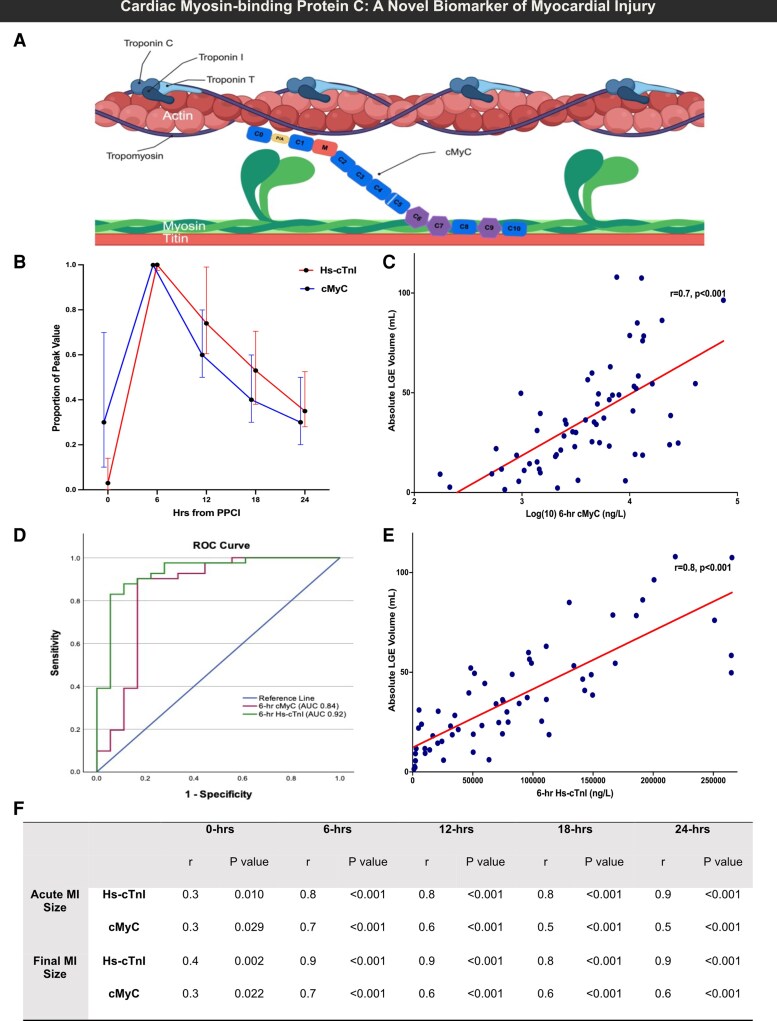
Cardiac myosin-binding protein C (cMyC): a novel biomarker of myocardial injury. Panel A: Structure of cMyC and cardiac troponins and their position within the sarcomere. Panel B: Graph demonstrating median hs-cTnI and cMyC at 6-hourly timepoints post PPCI for 24-hrs expressed as a proportion of the peak value. hs-cTnI and cMyC both peaked at 6-hrs post PPCI; cMyC fell more rapidly than hs-cTnI. Panels C and E: cMyC significantly correlated with acute and final MI size. Correlations between 6-hr hs-cTnI and cMyC and acute MI size are demonstrated. Panel D: cMyC demonstrated significant discriminatory power in identifying patients with acute MVO. ROC curves for 6-hr hs-cTnI and cMyC are shown. Panel F: Table demonstrating correlation coefficients (r) between hs-cTnI and cMyC at all timepoints and acute and final MI size. Amongst those with acute CMR data, biomarker analyses were analysed at 0-hrs (n = 69 for hs-cTnI, n = 68 for cMyC), 6-hrs (n = 62), 12-hrs (n = 67), 18-hrs (n = 69), and 24-hrs (n = 69). Amongst those with follow-up CMR data, biomarker data were reported at 0-hrs (n = 65 for hs-cTnI, n = 64 for cMyC), 6-hrs (n = 59), 12-hrs (n = 63), 18-hrs (n = 65), and 24-hrs (n = 65).

Amongst 82 recruited patients, blood samples were collected at the time of arterial sheath insertion (n = 80) and post-PPCI at 6-hrs (n = 72), 12-hrs (n = 78), 18- and 24-hrs (both n = 80) for biomarker analyses. One patient was excluded as cMyC was above the upper LoD for all timepoints. One patient had insufficient serum for baseline cMyC analysis. Acute and follow-up CMR were completed at 4 [4–5] days (n = 69) and 116 [113–122] days (n = 65), respectively, post PPCI. Four patients were excluded at follow-up due to arrhythmia (n = 1) and COVID-19 concerns (n = 3). The final numbers of hs-cTnI and cMyC assays reported in those with acute and follow-up CMR are summarized in Figure 1 legend, panel F.

Baseline clinical characteristics of patients with acute CMR data are shown in Table 1. The median [IQR] age was 56 [51–67] years; 90% were male. Median acute infarct volume was 34 [17–51] mL and 54% had anterior MI. The median pain-to-balloon time was 209 [156–423] minutes. Hs-cTnI [r =0.5 (P < 0.001)] and cMyC [r =0.3 (P = 0.009)] at baseline correlated with pain-to-balloon time.

**Table 1:** Baseline characteristics of patients with acute CMR data

	Patients with acute CMR (n = 69)
Age, years	56 [51–67]
Male sex, n (%)	62 (90)
**Risk factors, n (%)**	
Hypertension	28 (41)
Hypercholesterolaemia	43 (62)
Diabetes mellitus	17 (25)
History of smoking	41 (59)
Previous angina	19 (28)
**Presenting characteristics**	
Heart rate, beats/min	80 [67–90]
Systolic blood pressure, mmHg	139 [120–158]
Killip class, n (%)	
I	65 (94)
II	4 (6)
III or IV	0 (0)
GRACE risk score	98 [78–119]
TIMI risk score	2 [1–4]
Time from onset of chest pain to balloon inflation, min	209 [156–423]
**Culprit coronary artery, n (%)**	
Left anterior descending	37 (54)
Left circumflex artery	9 (13)
Right coronary artery	23 (33)
**TIMI coronary flow grade pre-PPCI, n (%)**	
0/1	52 (75)
2	10 (15)
3	7 (10)
**TIMI coronary flow grade post-PPCI, n (%)**	
0/1	0 (0)
2	5 (7)
3	64 (93)
**Medications on discharge, n (%)**	
Aspirin	68 (99)
P2Y12 inhibitor	69 (100)
Beta blocker	68 (99)
Angiotensin-converting enzyme inhibitor or angiotensin receptor blocker	67 (97)
Mineralocorticoid receptor antagonist	3 (4)
Statin	69 (100)

Values expressed as median [IQR], or number of patients (%).

Hs-cTnI and cMyC values rose from baseline at 712 [102–5662] ng/L and 812 [184–2950] ng/L, respectively, and peaked at 6-hrs post PPCI to 70 474 [22 006–125 993] ng/L and 4239 [1450–10 953] ng/L, respectively, before falling. cMyC fell more rapidly than hs-cTnI (Figure 1, panel B). Hs-cTnI and cMyC at all timepoints significantly correlated with acute and final MI size by absolute LGE volume (Figure 1, panel F). With increasing MI size by CMR, hs-cTnI and cMyC at all timepoints significantly increased. Hs-cTnI correlated more strongly with acute MI size compared with cMyC at 6-, 12-, 18-, and 24-hr timepoints and with final MI size at all timepoints. Correlations between hs-cTnI and acute MI size were strongest at the 24-hr timepoint (r = 0.9, P < 0.001) and for final MI size at 6-, 12-, and 24-hr timepoints (all r = 0.9, P < 0.001). By contrast, correlations for cMyC were strongest at the 6-hr timepoint for acute and final MI size (both r = 0.7, P < 0.001). Hs-cTnI at all timepoints and cMyC at 6-, 12-, 18-, and 24-hr post PPCI (AUC 0.84, P < 0.001; 0.78, P < 0.001; 0.76, P < 0.001; and 0.74, P = 0.002, respectively) demonstrated significant discriminatory power in identifying patients with acute MVO. The highest AUC for identifying MVO was observed at the 24-hr timepoint for hs-cTnI (AUC 0.94, P < 0.001) and at the 6-hr timepoint for cMyC (AUC 0.84, P < 0.001) (Table 2). Hs-cTnI had significantly higher discriminatory ability in identifying MVO at 12-, 18-, and 24-hr timepoints, compared with cMyC.

**Table 2:** AUC for acute serum biomarkers for identification of acute MVO

Acute serum biomarker^a^	AUC	95% CI	P value	Threshold (ng/L)	Sensitivity (%)	Specificity (%)
0-hr Hs-cTnI	0.66	0.52–0.80	0.033	>617	66	64
6-hr Hs-cTnI	0.92	0.85–1.00	<0.001	>50 371	84	89
12-hr Hs-cTnI	0.93	0.86–1.00	<0.001	>25 018	87	91
18-hr Hs-cTnI	0.93	0.86–1.00	<0.001	>21 988	85	91
24-hr Hs-cTnI	0.94	0.89–0.99	<0.001	>16 757	87	91
0-hr cMyC	0.61	0.47–0.76	0.139			
6-hr cMyC	0.84	0.71–0.97	<0.001	>2388	88	79
12-hr cMyC	0.78	0.65–0.92	<0.001	>1275	91	68
18-hr cMyC	0.76	0.62–0.90	<0.001	>741	94	64
24-hr cMyC	0.74	0.59–0.88	0.002	>835	87	64

^a^MVO was present in 47 patients (68%) amongst those with acute CMR data and biomarker data for baseline hs-cTnI and at 18- and 24-hrs (all n = 69). MVO occurred in 46 patients (68%) amongst those with acute CMR data and data for baseline cMyC (n = 68), and in 43 (69%) and 45 (67%) patients, respectively, in those with biomarker data at 6- and 12-hrs (n = 62 and n = 67, respectively).

## Discussion

This is the first demonstration that cMyC is a quantitative biochemical biomarker of acute and final MI size and predictor of acute MVO after STEMI, particularly at the 6-hr timepoint, when benchmarked against LGE-CMR. The strength of correlation with MI size and ability to predict MVO declines with increasing sampling intervals for cMyC, whilst increasing for hs-cTnI. This observation likely relates to the rapid release and clearance kinetics of cMyC.^1^ cMyC holds great promise for early diagnosis of acute MI, having previously been shown to identify a greater proportion of patients with acute MI in very early presenters compared with hs-cTnT.^7^ Nevertheless, in this report, hs-cTnI demonstrates a stronger correlation with MI size and higher discriminatory power in identifying MVO.^8^ These observed differences between hs-cTnI and cMyC may be explained in part by the use of an optimized commercial assay for the former report,^7^ compared with a prototype in-house developed non-commercial cMyC assay in this study, which has a smaller dynamic range than commercial platforms (Erenna®, EMD Millipore Corporation, Hayward, California).^7,9,10^ Furthermore, given its rapid release kinetics, cMyC may have peaked between baseline and the 6-hr timepoints. It is theoretically conceivable that cMyC may be more responsive to reperfusion than hs-cTnI. Further investigation with increased sampling frequency between 0 and 6 hrs post-PPCI is needed for clarification.

In conclusion, our results confirm hs-cTnI as the current reference standard for biochemical estimation of MI size and predictor of MVO. These results additionally suggest that cMyC is a promising biochemical marker of myocardial injury in reperfused STEMI. Further studies are warranted to confirm these results, in particular using other available optimized assay platforms,^7,9,10^ to further evaluate the performance of cMyC as a novel biomarker across the spectrum of acute myocardial ischaemic syndromes.^11^

## Data Availability

The data underlying this article will be shared on reasonable request to the corresponding author.
